# Evaluation of transabdominal ultrasound after oral administration of an echoic cellulose-based gastric ultrasound contrast agent for gastric cancer

**DOI:** 10.1186/s12885-015-1943-0

**Published:** 2015-11-25

**Authors:** Zhijun Liu, Jintao Guo, Shupeng Wang, Ying Zhao, Jing Li, Weidong Ren, Shaoshan Tang, Limei Xie, Ying Huang, Siyu Sun, Liping Huang

**Affiliations:** Ultrasound Department, Sheng Jing Hospital of China Medical University, No. 36 Sanhao Street, Shenyang, Liaoning Province 110004 People’s Republic of China; Endoscopy Center, Sheng Jing Hospital of China Medical University, Shenyang, Liaoning Province People’s Republic of China; Surgical Department, Sheng Jing Hospital of China Medical University, Shenyang, Liaoning Province People’s Republic of China

**Keywords:** Cellulose-based ultrasound contrast agent, Detection rate, Gastric cancer, Initial screening, Transabdominal ultrasound

## Abstract

**Background:**

With the remarkable improvements in ultrasound equipment, transabdominal ultrasound after oral administration of an echoic cellulose-based gastric ultrasound contrast agent (TUS-OCCA) has recently been suggested to be effective in initial screening of gastric cancer. The aim of this study was to evaluate the diagnostic value of TUS-OCCA for gastric cancer.

**Methods:**

Consecutive patients with gastric cancers who underwent resection in our hospital were enrolled. Before the lesion was resected, TUS-OCCA examination was performed by a skilled examiner who was blinded to the site, size, and endoscopy diagnosis of the lesion. TUS-OCCA findings were compared with those of endoscopy and pathological diagnoses as the gold standard.

**Results:**

There were a total of 288 consecutive patients enrolled in the study, including 228 with advanced gastric cancers (T2–T4 stage), 50 with early gastric cancer (26 with stage T1b and 24 with stage T1a), and 10 with high-grade intraepithelial neoplasia. TUS-OCCA had a detection rate of 100 % (228/228) for advanced gastric cancers, 77 % (20/26) for stage T1b, 67 % (16/24) for stage T1a, and 60 % (6/10) for high-grade intraepithelial neoplasia. The majority of patients with undetectable neoplasms using TUS-OCCA were obese (body mass index, 28.7–31.8 kg/m^2^). The overall accuracy of TUS-OCCA in determining the T stage of gastric cancer was 77.3 % (62.5 % for T1a, 70 % for T1b, 71.1 % for T2, 85.2 % for T3, and 73.3 % for T4).

**Conclusions:**

These findings indicate that TUS-OCCA achieved a high detection rate for gastric cancers and was useful in assessing the degree of gastric cancer invasion.

## Background

Gastric cancer is the fourth most common cancer and the second leading cause of cancer-related death worldwide [1–3]. The use of gastroscopy for opportunistic screening of gastric cancers is widely accepted; however, the employment of this procedure for mass screening of gastric cancers remains questionable, even in developed countries such as Japan [1–3]. Therefore, the barium swallow test continues to be the most common mass screening tool for gastric cancers in Japan and Korea [1–3]. However, a simple, economic, efficient, and noninvasive approach for mass screening of gastric lesions would be welcome.

Transabdominal ultrasound is increasingly used for the detection and evaluation of gastrointestinal lesions [4–11]. However, the diagnostic value of transabdominal ultrasound after oral administration of an echoic cellulose-based gastric ultrasound contrast agent (TUS-OCCA) for gastric cancer remains unclear. The purpose of this study was to evaluate the diagnostic value of TUS-OCCA for this disease.

## Methods

### Permission

The study protocol was approved by the Ethics Committee of Sheng Jing Hospital, China Medical University (Liaoning, China), and informed consent was obtained from all patients prior to enrollment.

### Patients

**Fig. 1:**
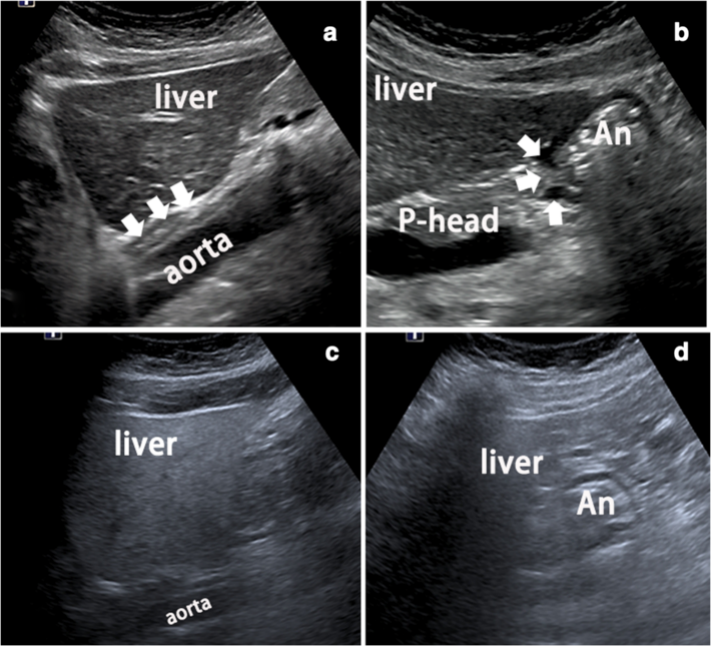
Definition of Groups S and U. **a** Visualization of the cardia (*arrows*) between the liver and aorta is acceptable. **b** Visualization of the pylorus (*arrows*) between the liver and pancreas is also acceptable. **c** The cardia between the liver and aorta is not acceptably visualized. **d** The pylorus between the liver and pancreasis is not acceptably visualized. Visualization of the cardia and pylorus is acceptable in Group S, but not in Group U. An, antrum; P-head, pancreatic head

From May 1, 2012 to June 1, 2015, consecutive patients who underwent resection in our hospital for gastric cancer and high-grade intraepithelial neoplasia were prospectively enrolled in this study. All of the enrolled subjects underwent an endoscopic examination and biopsy in our hospital or an affiliated facility approximately 1 week before surgery. For subgroup analysis, the enrolled patients were classified into two groups according to body habitus: Group S (suitable body habitus) and Group U (unsuitable body habitus). Group S included patients considered suitable for TUS-OCCA (visualization of the cardia and pylorus, as shown in Fig. 1a and b), while Group U included patients deemed less suitable for TUS-OCCA (inability to visualize the cardia or pylorus, as shown in Fig. 1c and d). The body habitus characteristics of the enrolled patients were evaluated using conventional transabdominal ultrasound before oral administration of contrast agent. The patients were subsequently classified into Groups S and U before TUS-OCCA examination was performed.

### TUS-OCCA examination

All TUS-OCCA examinations were performed before lesion resection by an investigator with 5 years of experience (ZJL) who was blinded to the site, size, and endoscopic diagnosis of the lesion; however, he was aware of the presence of gastric lesions scheduled to be resected surgically or endoscopically. Sonographic examinations were performed using the Toshiba Aplio 400 (Toshiba Medical Systems Corporation, Tokyo, Japan), Hitachi 8500 (Hitachi, Ltd., Tokyo, Japan), or IU22 (Philips Healthcare, Bothell, WA, USA) systems with a 2–5 MHz convex array probe.

### Cellulose-based oral contrast agent

The ultrasound contrast agent used was the World instant gastrointestinal ultrasound agent (Huzhou East Medical Devices, Huzhou, Zhejiang, China). It was reconstituted in 500 mL of boiling water, which formed a homogeneous thin paste. This was cooled to a suitable temperature and then administered orally to improve stomach distension. The uniform thin paste formed by this cellulose-based contrast agent reportedly had a pleasant taste (slightly sweet) and was well tolerated by most patients. TUS-OCCA examination was performed at approximately 1 min after the contrast agent was swallowed by the patient. The acoustic velocity and specific acoustic impedance of the contrast agent were similar to those of liver tissue. The stomach, once filled with the cellulose-based ultrasound contrast agent, appeared as a homogeneous mid- to high-level echogenicity. No antispasmodics were used.

### Stomach scanning procedure

**Fig. 2:**
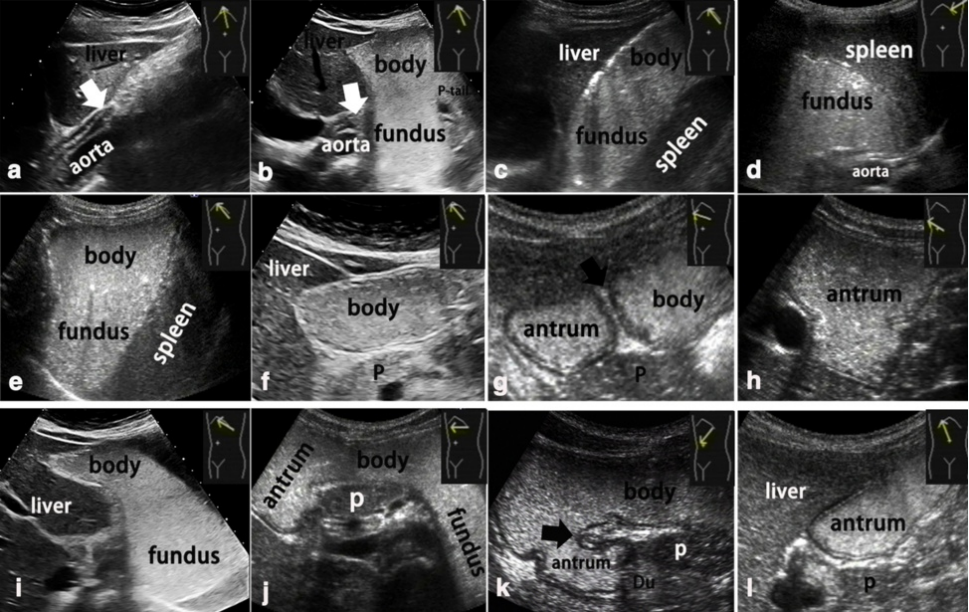
Normal sections of the contrast-filled whole stomach obtained in five views: (1) sagittal section of the **a** lower esophagus (*white arrow*), **b** gastric cardia (*white arrow*), and **c** oblique section of the gastric fundus (cardia; *white arrow*); (2) **d** transverse section of the gastric fundus; (3) serial transverse sections of **e** gastric fundus, **f** body, **g** angle, and **h** antrum (*black arrow*; transverse section of the gastric angle); (4) serial coronal sections of **i** gastric fundus, **j** body, **k** angle and antrum (*black arrow*; coronal section of the gastric angle); and (5) **l** longitudinal section of the gastric antrum. P, pancreas

The whole stomach was scanned in five views as follows (Fig. 2): (1) mainly the gastric cardia (Fig. 2a and b) and then the gastric fundus (Fig. 2c), which was performed by moving the probe from the xiphoid process to the left costal arch, with the subject in the supine position; (2) mainly the gastric fundus (Fig. 2d), which was performed by placing the probe at the location of the left intercostal space; (3) the gastric fundus, body, and antrum in a transverse section (Fig. 2e–h), which was performed by moving the probe from the left costal arch to the right costal arch, with the subject lying in the right decubitus position; (4) the gastric fundus, body, and antrum in a coronal section (Fig. 2i–k), which was performed by rotating the probe along the left costal arch, while using the caudal end of the probe as an axis (simultaneously tilting the probe at ~45°) with the subject lying in the right decubitus position; and (5) the gastric antrum (Fig. 2l), which was performed by placing the probe vertically to the right costal arch, with the subject lying in the supine position. Views 3 (Fig. 2e–h) and 4 (Fig. 2i–k) were key to obtaining serial transverse and coronal sections, respectively, of the whole stomach including the gastric fundus, body, angle, and antrum.

### Sonographic criteria

The sonographic criteria for the diagnosis of stomach neoplasms were based on recommendations in the literature [12, 13] and our previous experience. Gastric high-grade intraepithelial neoplasia and intramucosal (stage T1a) early gastric cancer presented on ultrasonograph scans as hypoechoic thickening of the mucosal layer, with the hyperechoic submucosal layer intact. Early gastric cancer involving the submucosal layer (stage T1b) presented as hypoechoic thickening of the mucosal and submucosal layers, with the hypoechoic muscularis propria layer intact. Advanced gastric cancers (stage T2–T4) presented as hypoechoic thickening of the gastric wall with disruption to the muscularis propria layer. Features, such as tumor size, site, and echo pattern, were recorded during each TUS-OCCA examination and the findings were compared with those of endoscopic and pathological diagnoses.

All statistical analyses were performed using the Fisher’s exact test with SPSS statistical software (version 21.0: IBM-SPSS, Inc., Chicago, IL, USA). A probability (*p*) value < 0.05 was considered statistically significant.

## Results

A total of 288 patients (178 men and 110 women; mean age, 54.6 years; age range, 32–83 years) who underwent treatment for gastric cancer and high-grade intraepithelial neoplasia were enrolled. Patients included 228 with advanced gastric cancers (stage T2–T4 stage), 50 with early gastric cancers (26 with stage T1b and 24 with stage T1a), and 10 with high-grade intraepithelial neoplasia. According to the habitus characteristics evaluated using transabdominal ultrasound, 184 (64 %) patients were classified into Group S and 104 (36 %) into Group U.

As shown in Table 1, TUS-OCCA achieved a detection rate of 100 % (228/228) for advanced gastric cancers, 77 % (20/26) for stage T1b early gastric cancer, 67 % (16/24) for stage T1a early gastric cancer, and 60 % (6/10) for high-grade intraepithelial neoplasia. Subgroup analysis of the detection rates for TUS-OCCA in the patients within Group S (146 patients with advanced gastric cancer, 32 patients with early gastric cancer, and 6 with high-grade intraepithelial neoplasia) was 100 % (184/184). The detection rates for TUS-OCCA in the patients in Group U were 83 % (86/104) (including 100 % (82/82) for advanced gastric cancers, 18 % (4/22) for early gastric cancer and high-grade intraepithelial neoplasia). The 14 patients with early gastric cancer and four with high-grade intraepithelial neoplasia who were undetected using TUS-OCCA examination were in Group U. The difference in the detection rates between Groups S and U (100 % vs. 83 %) was statistically significant (*p* < 0.05, Fisher’s exact test).

**Table 1:** Detection rates for gastric cancer and high-grade intraepithelial neoplasia using TUS-OCCA

Gastric cancer and premalignant lesions	Detection rates		Total
	Group S	Group U	
High grade intraepithelial neoplasia	6/6	0/4	6/10 (60 %)
Stage T1a early gastric cancer	14/14	2/10	16/24 (67 %)
Stage T1b early gastric cancer	18/18	2/8	20/26 (77 %)
Advanced gastric cancer (stage T2 ~ T4)	146/146	82/82	228/228 (100 %)
Total	184/184 (100 %)	86/104 (83 %)	270/288 (94 %)

TUS-OCCA, transabdominal ultrasound after oral administration of an echoic cellulose-based contrast agent. The difference in the detection rates between Groups S and U (100 % vs. 83 %) was significant (*p* < 0.05, Fisher’s exact test)

Subgroup analysis data regarding the detection rates using TUS-OCCA evaluated according to the location of the lesions are detailed in Table 2. For lesions located in the antrum, the detection rate was 95 % (87/92); for lesions located in the angle, the detection rate was 96 % (70/73); for lesions located in the body, the detection rate was 97 % (38/39); for lesions located in the fundus, the detection rate was 63 % (5/8); and for lesions located in the cardia, the detection rate was 92 % (70/76).

**Table 2:** Detection rates for gastric cancer and high-grade intraepithelial neoplasia using TUS-OCCA (data analyzed according to location)

Location of lesions	Patients (n)	Detection rates of TUS-OCCA
Antrum	92	87/92 (95 %)
Angle	73	70/73 (96 %)
Body	39	38/39 (97 %)
Fundus	8	5/8 (63 %)
Cardia	76	70/76 (92 %)
Total	288	270/288 (94 %)

TUS-OCCA, transabdominal ultrasound after oral administration of an echoic cellulose-based gastric ultrasound contrast agent

**Fig. 3:**
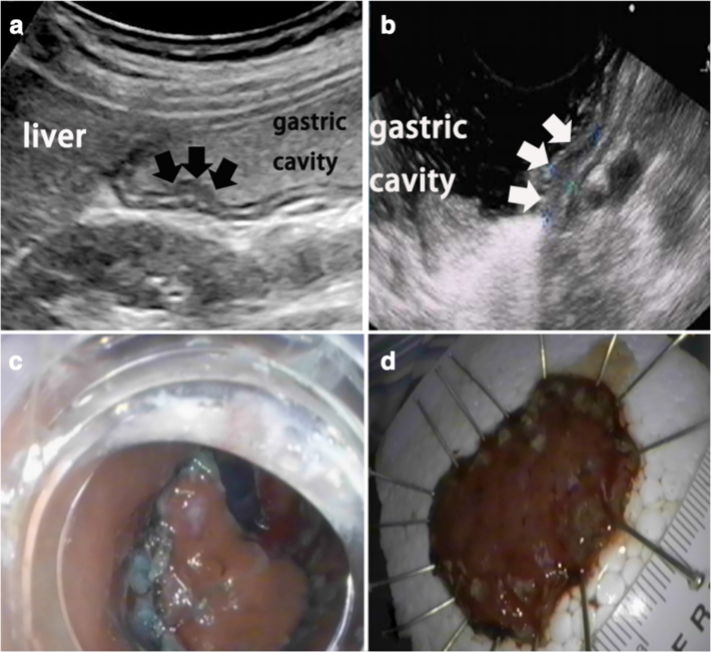
Early gastric cancer. **a** Examination involving transabdominal ultrasound scans after oral administration of an echoic cellulose-based gastric ultrasound contrast agent showing hypoechoic mucosal thickening of the gastric wall (*black arrows*). **b** Endoscopic ultrasound showing hypoechoic mucosal thickening of the gastric wall (*white arrows*). **c** and **d** Endoscopic resection of a lesion confirmed as early gastric cancer

**Fig. 4:**
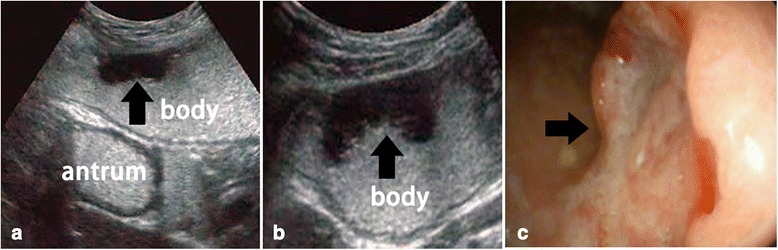
Advanced gastric cancer. **a** and **b** Examination involving transabdominal ultrasound scans after oral administration of an echoic cellulose-based gastric ultrasound contrast agent showing hypoechoic thickening of the gastric wall with superficial ulceration (a, longitudinal section; b, cross section). **c** Gastroscopic examination showing the lesion. Arrow, pointing at the lesion

As detailed in Table 3, the overall accuracy of TUS-OCCA in determining the T stage of gastric cancer was 77.3  % (62.5  % for T1a, 70  % for T1b, 71.1  % for T2, 85.2  % for T3, and 73.3  % for T4). Figures 3 and 4 show examples of early and advanced gastric cancers, respectively on TUS-OCCA images.

**Table 3:** Results of T staging using TUS-OCCA as compared with the pathological findings (by case)

Pathology	TUS-OCCA					Accuracy
	T1a	T1b	T2	T3	T4	
T1a	10	6	0	0	0	62.5 % (10/16)
T1b	2	14	4	0	0	70.0 % (14/20)
T2	0	4	54	18	0	71.1 % (54/76)
T3	0	0	12	104	6	85.2 % (104/122)
T4	0	0	0	8	22	73.3 % (22/30)

TUS-OCCA, transabdominal ultrasound after oral administration of an echoic cellulose-based ultrasound contrast agent. The overall accuracy of TUS-OCCA for T-staging was 77.3 % (204/264)

## Discussion

Because gastric cancer is the second leading cause of cancer-related death worldwide, it is important in terms of the further development of anticancer strategies [14–16]. The 5-year survival rate for gastric cancers at stages IA, IB, II, IIIA, IIIB, and IV are reportedly 92.2, 85.3, 72.1, 52.8, 31.0, and 14.9 %, respectively [17]. Hence, earlier detection of gastric cancers will enable better prognosis.

Various imaging modalities are used to detect gastric lesions, including endoscopy, barium studies, computed tomography, magnetic resonance imaging, and ultrasound [18–21]. Nevertheless, to date, no suitable mass screening tool for gastric cancer has been recommended by the World Health Organization; hence, there is a need for a method that is relatively safe, simple, inexpensive, and reliable [22]. Gastroscopy appears to be the most accurate method for the detection of gastric lesions; however, patient discomfort, risk of cross-infection, the high cost of screening, and the lack of experienced endoscopists hamper its application. Therefore, barium swallow continues to be the main choice for mass screening of gastric cancer in Japan and Korea [1–3]. In Japan, approximately 4,000,000 individuals undergo barium swallow testing annually, and about 10 % of those with abnormal results are further screened using endoscopy [2]. Moreover, an additional 1,000,000 individuals in Korea undergo barium studies annually [3]. However, the barium swallow test is an observational approach and its ability is somewhat limited. In addition, barium studies are quite invasive in terms of complications, especially constipation and mis-swallowing of the barium into the trachea [23]. In consideration of these complications, a simple, economic, efficient, and noninvasive approach for mass screening of gastric cancer would be welcome.

**Fig. 5:**
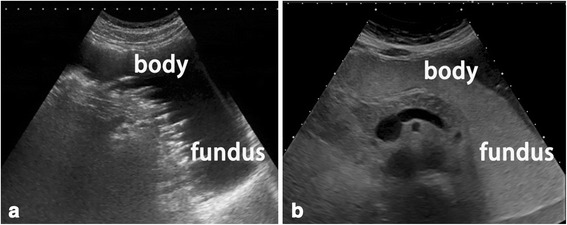
Cellulose-based contrast agent may provide fewer gas artifacts than water. **a** Mural gas and gastric mucus adherence to the gastric wall was increased when the stomach was filled with water, which may influence observations of the gastric wall. **b** Mural gas and gastric mucus adherence to the gastric wall were reduced when the stomach was filled with cellulose-based contrast agent, as a result of its greater bulk and surface tension properties, which optimize gas displacement and interfere with mural gas adherence

**Fig. 6:**
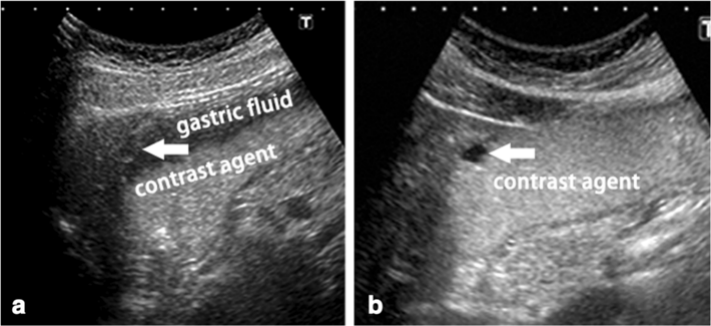
As compared with water, the uniformity of the mid to high echoic contrast agent may improve ultrasound visualization of hypoechoic gastric tumors. **a** Because the echo intensity of the tumor was similar to that of the gastric fluid floating above the cellulose-based contrast agent, visualization of the hypoechoic tumor was blurred and unclear. **b** The uniformity of the mid to high echoic contrast agent could improve ultrasound visualization of the hypoechoic gastric tumors. Arrow, pointing at the hypoechoic tumor

With the remarkable improvements in ultrasound equipment, TUS-OCCA has recently been suggested by some authors to be effective in initial gastric cancer screening for selected individuals [4–7]. The reported benefits of the mid-high echoic cellulose-based gastrointestinal contrast agent include the following [4–6, 24]: (1) fewer gas artifacts than water because of the superior bulk and surface tension properties, which optimize gas displacement and interfere with mural gas adherence (Fig. 5a and b); (2) the uniform mid to high level of echogenicity provided by this cellulose-based contrast agent can improve the ultrasonic visualization of hypoechoic gastric lesions (Fig. 6a and b); and (3) small gastric lesions can be detected much easier because gastric mucosal folds are more effectively flattened by this thin paste cellulose-based contrast agent.

Some previous comparative studies have reported that TUS-OCCA was remarkably accurate in the detection of various gastric lesions, with sensitivities of 77.8–100 % and specificities of 94–100 % [4–6]. Li et al. [7] clearly demonstrated the morphological features of 350 gastric lesions using transabdominal ultrasonography, including 53 located in the cardia, 70 in the fundus, 90 in the body, 99 in the antrum, and 38 in the pylorus. Shi et al. [8] reported that transabdominal ultrasound clearly demonstrated the anatomy of the stomach and the morphological features of various gastric lesions in all 46 patients evaluated, including 5 with chronic gastritis, 9 with gastric ulcers, 3 with gastric polyps, 5 with gastric stromal tumors, 24 with gastric cancers, and 1 with postoperative recurrent gastric cancer.

The ability of TUS-OCCA to detect gastric lesions is influenced by the body habitus, as well as the location, size, and echogenicity of the gastric lesion. Body habitus is an important influential factor. As detailed in Table 1, the detection rate for stomach neoplasms using TUS-OCCA in Group S was greater than that in Group U (100 % vs. 83 %). Therefore, TUS-OCCA is strongly suggested for selected individuals, especially those considered non-obese with a suitable body habitus (visualization of the cardia and pylorus) [4–6]. As is known, conventional transabdominal ultrasound has been widely used for population-based mass screening of abdominal cancers (such as liver, gallbladder, pancreas, kidney, bladder, and others). An individual’s body habitus can be assessed during conventional transabdominal ultrasound examination [6]. For individuals with a suitable body habitus, TUS-OCCA examination could then be strongly suggested for gastric cancer screening [6].

Location is also an important influential factor. The lesions located in the gastric antrum and body were much easier to detect than those in the fundus (Table 2); the detection rate achieved using TUS-OCCA for lesions located in the fundus was relatively low (only 63 %). Because the fundus is situated relatively deeply in the body (usually at depths >15 cm) during transabdominal ultrasound scanning from the left costal arch and left intercostal space, the ultrasound imaging of far field of the fundus is blurred [4–7]. In addition, scanning of near field of the fundus would be hampered by the costal bone when locating the probe from the left intercostal space. In other words, unlike the antrum and the body, scanning of the whole fundus is not very satisfactory [4–7]. Fortunately, gastric cancer located in the fundus is rare. As has been established, the majority of gastric malignant neoplasms and precancerous lesions infiltrate the gastric lesser curvature (including pylorus, angle, and cardia) and antrum, rather than the gastric fundus [25, 26].

The echogenicity of lesions is another important influential factor. In our experience, the detection rates of TUS-OCCA for gastric polyps, which usually present with a mid to high echo, are low; the detection rates of TUS-OCCA for gastric cancer, which usually presents as hypoechoic thickening of the gastric wall, are high.

The most important reason why TUS-OCCA is currently performed and continues to be investigated is that the pathological regression of various gastric lesions has been better elucidated over the past 5 years. In defense of this position, the following factors should be considered: (1) the majority of gastric polyps are benign and are believed to possess no malignant potential [27–30]; (2) gastric precancerous lesions are usually composed of gastric mucosa with intestinal metaplasia or dysplasia presenting as hypoechoic mucosal thickening [31, 32]. Although the detection rate using TUS-OCCA for gastric polyps (which usually require no further treatment) is low, the detection rate for hypoechoic thickening of the gastric wall (which requires further treatment) is high.

One limitation of our study in common with others was that the gastric lesions of the enrolled patients were previously detected in gastroscopic examinations; this may have influenced the higher accuracy rates for the detection of the lesions, even though the examiner was blinded to the site, size, and endoscopic diagnosis of the lesions. Another limitation of the study is that it lacked negative controls, which also limited its accuracy.

In our experience, the sensitivity and specificity of TUS-OCCA for gastric dysplasia and cancer were 90.8 and 96 %, respectively [6]. The majority of patients whose lesions were undetected using TUS-OCCA were obese, and were classified into Group U [6]. In our experience, about 70 % of the population would be classified into Group S and about 30 % would be classified into Group U. Although TUS-OCCA examination is not suggested by ultrasonic physicians for patients classified into Group U, many still want to simultaneously undergo TUS-OCCA for mass screening of gastric cancer when they undergo conventional transabdominal ultrasound examination for mass screening of abdominal cancer. Certainly, written informed consent should be obtained from patients after they have been advised regarding the limits and disadvantages of TUS-OCCA. We believe that with promotion of the TUS-OCCA technique and training in its use, it could be used as an alternative mass screening tool for gastric cancers, especially in the Asian population (China, Japan, and Korea) where the incidence of the disease is high and the body habitus of the population is more suitable.

## Conclusions

TUS-OCCA achieved a high detection rate for gastric cancers, especially in patients with a suitable body habitus whose cardia and pylorus could both be visualized using transabdominal ultrasound. TUS-OCCA could be used to assess the depth of gastric cancer invasion. Many more successive studies will be needed to fully evaluate the utility of TUS-OCCA.
